# Supplementary material for: Vitreomacular Interface Disorders in Proliferative Diabetic Retinopathy: An Optical Coherence Tomography Study
Source: J Clin Med. 2022 Jun 7;11(12):3266. doi: 10.3390/jcm11123266 (PMC9224563; doi:10.3390/jcm11123266)
Supplement: Supplementary file 1 [file jcm-11-03266-s001.zip › jcm-1660495-supplementary.pdf]

Table S1. Kappa statistics for the interobserver concordance of the OCT evaluation.

OCT findings	Kappa (95% CI)
VMA	0.994 (0.988-1.000)
VMT	0.938 (0.924-0.952)
ERM	0.987 (0.979-0.995)
LHEP	0.993 (0.987-0.999)
LMH	0.992 (0.986-0.998)
FTMH	0.999 (0.997-1.001)
macular cysts	0.984 (0.976-0.992)
retinoschisis	0.982 (0.974-0.990)
RD	0.958 (0.946-0.970)
EZ disruption	0.924 (0.908-0.940)
DRIL	0.901 (0.883-0.919)
hard exudates	0.931 (0.915-0.947)

Abbreviations: OCT, optical coherence tomography; VMA, vitreomacular adhesion; VMT, vitreomacular traction; ERM, epiretinal membrane; LHEP, lamellar hole-associated epiretinal proliferation; LMH, lamellar macular hole; FTMH, full-thickness macular hole; RD, retinal detachment; EZ, ellipsoid zone; DRIL, disorganization of retinal inner layers; 95% CI, 95% confidence interval
